# 

**Published:** 1912-10-05

**Authors:** 


**The Modern Newspaper of Administrative — Medicine and Institutional Life.**

Telegraphic Address: OSPEDALE, RAND, LONDON. Telephone Number: 2734 GERRARD.

No. 1370, Vol. LIII. | Saturday, October 5th, 1912. | PRICE ONE PENNY

